# Estimation of Incubation Period for Oropouche Virus Disease among Travel-Associated Cases, 2024–2025

**DOI:** 10.3201/eid3107.250468

**Published:** 2025-07

**Authors:** Sarah Anne J. Guagliardo, Stacey Martin, Carolyn V. Gould, Rebekah Sutter, Daniel Jacobs, Kevin O’Laughlin, Ralph Huits, Concetta Castilletti, J. Erin Staples, GeoSentinel Network Collaborators

**Affiliations:** Author affiliations: Centers for Disease Control and Prevention, Fort Collins, Colorado, USA (S.A.J. Guagliardo, S. Martin, C.V. Gould, R. Sutter, D. Jacobs, J.E. Staples); Centers for Disease Control and Prevention, Atlanta, Georgia, USA (K. O’Laughlin); IRCCS Sacro Cuore Don Calabria Hospital, Verona, Italy (R. Huits, C. Castilletti)

**Keywords:** Oropouche, viruses, vector-borne infections, incubation period, interval-censored survival analysis, United States

## Abstract

Determining the incubation period of Oropouche virus disease can inform clinical and public health practice. We analyzed data from 97 travel-associated cases identified by the Centers for Disease Control and Prevention (n = 74) or the GeoSentinel Network (n = 13) and 10 cases from published literature. Using log-normal interval-censored survival analysis, we estimated the median incubation period to be 3.2 (95% CI 2.5–3.9) days. Symptoms developed by 1.1 (95% CI 0.6–1.5) days for 5% of patients, 9.7 (95% CI 6.9–12.5) days for 95% of patients, and 15.4 (95% CI 9.6–21.3) days for 99% of patients. The estimated incubation period range of 1–10 days can be used to assess timing and potential source of exposure in patients with Oropouche symptoms. For patients with symptom onset >2 weeks after return from travel, clinicians and public health responders should consider the possibility of local vectorborne transmission or alternative modes of transmission.

Oropouche virus disease (Oropouche) is caused by infection with Oropouche virus (OROV; genus *Orthobunyavirus*, Simbu serogroup). In late 2023, a large outbreak of Oropouche originated in the Brazilian Amazon, later expanding into endemic and nonendemic regions in the Americas, and >16,000 cases were reported by the end of 2024 (*1*). Since 2023, >140 cases have been identified in travelers returning to Europe and North America, predominantly from Cuba (*2*).

Clinical manifestations of Oropouche are similar to those of other vectorborne diseases, such as dengue, Zika, and chikungunya, and are characterized by acute onset of fever, headache, myalgia, fatigue, chills, and arthralgia (*3*). Other symptoms can include diarrhea, nausea, vomiting, maculopapular rash, abdominal pain, retroorbital pain, back pain, and photophobia (*4*). Although most Oropouche cases are mild, severe disease and death have been reported (*5*). Severe manifestations of illness include hemorrhagic symptoms (e.g., gingival bleeding, melena, and menorrhagia), neurologic symptoms (e.g., meningitis, meningoencephalitis, Guillain-Barré syndrome), and adverse pregnancy outcomes (*6*–*9*).

OROV is primarily transmitted to humans through the bites of infected biting midges (*Culicoides paraensis*) and possibly certain mosquito species, such as *Culex quinquefasciatus.* Other observed transmission modes have included accidental inoculation via oral and respiratory routes in a laboratory setting (*10*). Oropouche viral RNA was recently detected in the semen of 2 travelers returning to Europe from Cuba, raising questions about the possibility of sexual transmission, although that mode of transmission has not yet been confirmed (*11*,*12*). Congenital transmission is also suspected because of reported maternal infections during pregnancy that resulted in birth defects and laboratory evidence of OROV infection in those infants (*9*).

The incubation period for Oropouche was previously estimated to range from 3 to 10 days, but that estimate was based on just 2 cases (*3*,*6*,*10*). Having a more precise estimation of the incubation period can help clinicians form a differential diagnosis on the basis of timing of potential exposures and help public health officials distinguish between travel-associated cases and local transmission. We estimated the Oropouche incubation period using data from infected travelers returning to nonendemic areas.

## Methods

### Data Sources

**Figure 1:**
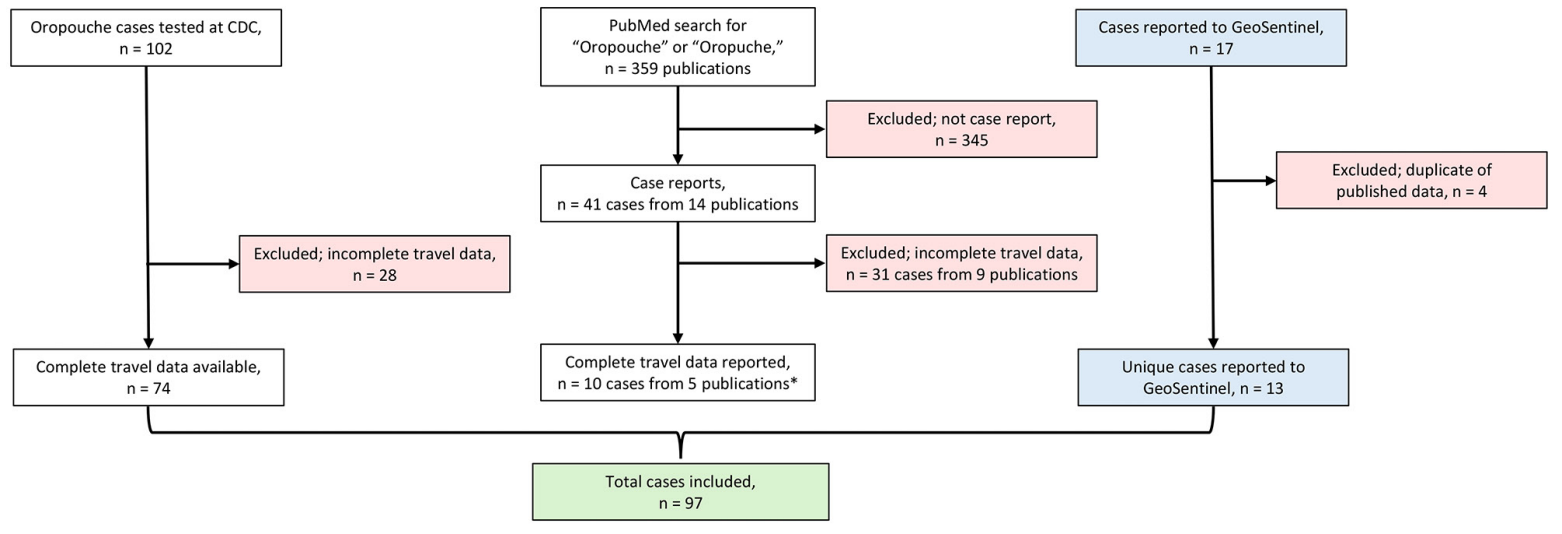
Flowchart of data sources and 97 cases included in an estimation of incubation period for Oropouche virus disease among travel-associated cases, 2024–2025. The study included patients who developed symptoms and had positive test results for Oropouche in the laboratory database of the CDC Arboviral Diseases Branch (Division of Vector-borne Diseases, National Center for Emerging and Zoonotic Infectious Diseases), through GeoSentinel (https://geosentinel.org), or in reports available on PubMed as of March 15, 2025. We excluded patients without complete travel data. *(*6*,*11*,*13*–*15*). CDC, Centers for Disease Control and Prevention.

We used 3 data sources: the laboratory database of OROV testing conducted during 2024–2025 through the Centers for Disease Control and Prevention (CDC) Arboviral Diseases Branch (Division of Vector-borne Diseases, National Center for Emerging and Zoonotic Infectious Diseases); the GeoSentinel Network database (https://geosentinel.org) of patients identified with Oropouche during 2024; and a PubMed search of the literature through March 15, 2025, using the search terms “Oropouche” or “Oropuche” (Figure 1). At CDC, suspected cases were tested by real-time reverse transcription PCR (rRT-PCR) or 90% plaque reduction neutralization test, depending on the timing of specimen collection relative to symptom onset (*16*). The GeoSentinel Network, a collaboration between CDC and the International Society of Travel Medicine, is a global surveillance system for illnesses affecting international travelers currently comprised of ≈70 clinical sites in 30 countries. Patients identified and tested through the GeoSentinel Network were tested for evidence of OROV infection by rRT-PCR or IgG and IgM serology. For each case-patient, we extracted basic demographic data (age and sex), symptom onset date, travel dates, and whether the patient was hospitalized. Patients self-reported symptom onset date and travel dates. We ensured no overlap among patients identified through those sources by verifying that demographic data and travel dates were unique.

### Case Classification and Inclusion Criteria

We included probable and confirmed cases of Oropouche. We defined a probable case as a patient with a known epidemiologic link whose blood or cerebrospinal fluid sample tested positive for OROV-specific IgM or neutralizing antibodies. We defined a confirmed case as a patient with a known epidemiologic link whose sample was OROV-positive by rRT-PCR, had a >4-fold change in neutralizing antibody titers in paired serum samples, or was positive for IgM in blood or cerebrospinal fluid with confirmatory virus-specific neutralizing antibodies. We only included symptomatic patients that had complete information about travel destinations, departure and return dates, and symptom onset date. We considered any of the symptoms included in the suspected case definition (e.g., fever, chills, headache, myalgia, arthralgia, retro-orbital pain, or generalized rash) to denote the onset of symptoms, as self-reported by patients.

### Statistical Analysis

**Figure 2:**
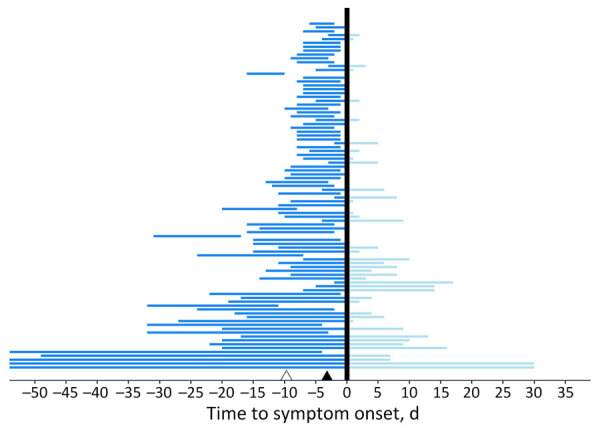
Time of exposure relative to symptom onset in an estimation of incubation period for Oropouche virus disease among 97 probable and confirmed travel-associated cases, 2024–2025. Each horizontal line corresponds to an individual patient’s exposure time. The vertical black line represents symptom onset. The horizontal lines represent the exposure durations before (dark blue) and after (light blue) symptom onset. Observations are ordered by duration of travel, and long travel durations are truncated from the graph for ease of interpretation. The black triangle represents the median incubation period for probable and confirmed cases; the white triangle represents the 95th quantile.

We conducted descriptive statistics on patient demographics (age and sex) and travel characteristics (travel duration and location). We used parametric interval-censored survival modeling to estimate the incubation period distribution (*17*,*18*). We determined the exposure period by using the dates of travel relative to the timing of illness onset. Specifically, for patients whose symptoms developed after travel, we considered the exposure period to be the duration of travel, and for patients whose symptoms developed during travel, we considered the exposure period to be from the beginning of travel through the illness onset date (Figure 2) (*17*,*18*). 

We fitted probability distributions (Weibull, log-normal, and Gamma) to the data by using the dic.fit function in the coarseDataTools package in R (The R Project for Statistical Computing, https://www.r-project.org) and selected the best-fitting distribution using the Akaike information criterion (*17*,*18*). We calculated cumulative distribution functions and associated 95% CIs, along with means, medians, and 5th, 95th, and 99th quantiles. We also evaluated whether estimated incubation period varied by age, sex, and hospitalization status by fitting parametric log-normal models for each covariate using the survreg procedure in the survival package in R.

The initial analysis included all patients that met inclusion criteria. To provide a more precise estimate of incubation periods, we also performed an analysis on a subset of patients meeting the case definition for confirmed Oropouche and who had <14 days of travel. Last, we analyzed a subset of cases detected in 2024–2025 to examine if the current viral strain affected the incubation period estimates.

### Ethics Considerations

This activity was reviewed by CDC, was deemed not research, and was conducted consistent with applicable federal law and CDC policy (project no. 0900f3eb824f7cc8). Applicable federal laws include 45 C.F.R. part 46.102(l) (2), 21 C.F.R. part 56; 42 U.S.C. Sect. 241(d); 5 U.S.C. Sect. 552a; 44 U.S.C. Sect. 3501 et seq. A human subjects advisor at CDC’s National Center for Emerging and Zoonotic Infectious Diseases reviewed the GeoSentinel surveillance data collection protocol and classified it as public health surveillance and not human subjects research (project no. 0900f3eb81bc3a03). Additional ethics clearance was obtained by GeoSentinel sites, as required by their respective institutions. 

## Results

In total, 97 cases met the inclusion criteria, consisting of 74 cases identified on the basis of testing conducted at CDC, 13 reported to GeoSentinel, and 10 identified in the peer-reviewed literature (Figure 1). The symptom onset dates ranged from October 2010 to January 2025, and 98% (n = 95) of cases occurred during 2024–2025.

Most (96%, n = 93) patients were adults >19 years of age; 54 (56%) were female and 43 (44%) were male (Table 1). More than half (57%, n = 55) of patients had initial symptoms develop during travel, and the median exposure period was 7 days (interquartile range [IQR] 6.0–14.0 days; range 2–135 days) (Figure 2). The most common travel location was Cuba, as reported by 97% (n = 94) of patients. The demographic characteristics of the confirmed and probable cases compared with confirmed cases with <14 days of travel were similar (Table 1). Compared with cases identified through GeoSentinel and published literature, patients whose samples were tested at CDC were older (median age 51 years for cases tested at CDC vs. 30 years for cases identified elsewhere; p<0.0001), less likely to have been hospitalized (16% vs. 40%; p = 0.041), and had shorter exposure periods (median 7 days vs. 14 days; p<0.0001) (Appendix Table 1).

**Table 1:** Characteristics of probable and confirmed cases used in an estimation of incubation period for Oropouche virus disease among travel-associated cases, 2024–2025*

Characteristics	Probable and confirmed, n = 97	Confirmed, n = 40†
Age group, y		
0–19	4 (4.1)	1 (2.5)
20–39	32 (33.0)	12 (30.0)
40–59	41 (42.3)	21 (52.5)
>60	20 (20.6)	6 (15.0)
Missing	0	0
Sex		
F	54 (55.7)	22 (55.0)
M	43 (44.3)	18 (45.0)
Missing	0	0
Hospitalized		
N	72 (78.3)	32 (80.0)
Y	20 (21.7)	8 (20.0)
Missing	5	0
Travel duration, d		
<7	21 (21.6)	11 (27.5)
7–13	38 (39.2)	25 (62.5)
14–20	17 (17.5)	4 (10.0)‡
21–27	8 (8.2)	NA
>28	13 (13.4)	NA
Missing	0	0
Onset during travel		
N	42 (43.3)	22 (55.0)
Y	55 (56.7)	18 (45.0)
M	0	0
Exposure period, d		
Mean	13.6	7.3
Median (IQR)	7 (6.0–14.0)	7 (6–9)
Range	2–135	2–14

*Values are no. (%) except as indicated. NA, not applicable. †Traveled <14 days. ‡Traveled for 14 days.

**Figure 3:**
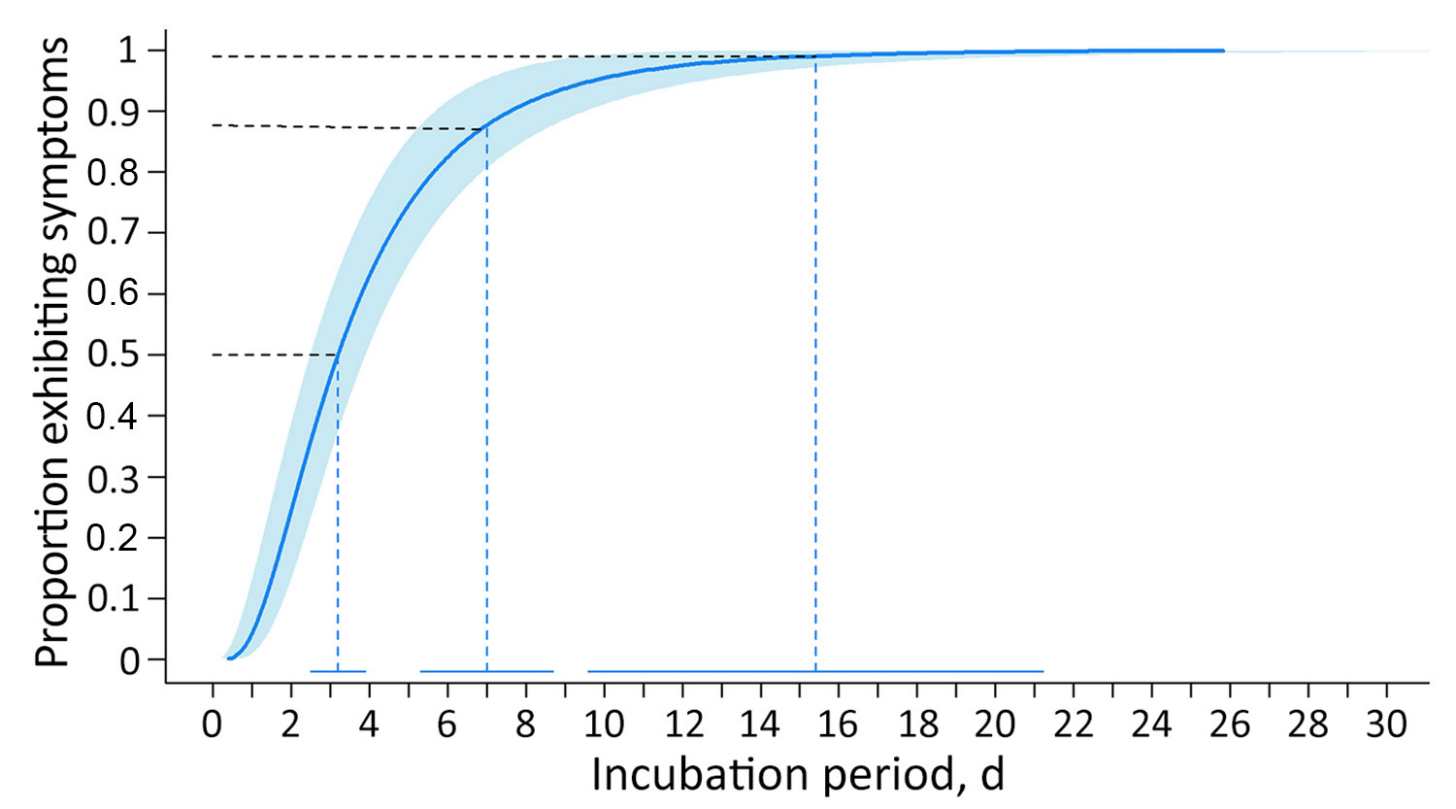
Estimated cumulative distribution of incubation period for Oropouche virus disease among 97 probable and confirmed travel-associated cases, 2024–2025. Solid blue line represents the estimated log-normal cumulative distribution function; shaded area represents 95% CI. Dashed lines correspond to the 50th and 99th quantiles, in addition to the proportion of patients that experienced symptoms within 1 week (88th quantile). The solid horizontal lines at bottom represent the 95% CIs for the quantiles.

The log-normal distribution was the best fit for all case sets (Appendix Table 2). For probable and confirmed cases (n = 97), the estimated median incubation period was 3.2 (95% CI 2.5–3.9) days (Table 2). We estimated symptoms developed within 1.1 (95% CI 0.6–1.5) days for 5% of patients and within 9.7 (95% CI 6.9–12.5) days for 95% of patients. We estimated that most (88%) patients’ symptoms developed within 7 days (Figure 3). Our estimates show that almost all (99%) patients had symptoms develop by 15.4 (95% CI 9.6–21.3) days. The estimated median incubation period for confirmed cases only was similar at 3.1 (95% CI 2.1–4.0) days, and 95% of confirmed cases had symptoms develop within 9.3 (95% CI 5.4–13.2) days (Appendix Table 3). Estimated incubation periods did not differ substantially by age, sex, hospitalization status, or when we excluded the 2 cases that occurred before 2024 (Appendix Tables 3, 4).

**Table 2:** Parameters and quantiles of log-normal distribution in an estimation of incubation period for Oropouche virus disease among 97 probable and confirmed travel-associated cases, 2024–2025

Parameter and quantile	Estimate (95% CI)
Location parameter, µ	3.2 (2.6–4.0)
Dispersion parameter, σ	2.0 (1.6–2.3)
Quantile	
5th	1.1 (0.6–1.5)
50th	3.2 (2.5–3.9)
95th	9.7 (6.9–12.5)
99th	15.4 (9.6–21.3)

## Discussion

In this analysis of travel-associated Oropouche cases, we estimated the median incubation period to be 3–4 days, with a range of 1 day (5% of cases) to 10 days (95% of cases). We estimated symptoms developed within 3 days of exposure for 50% of patients and within 15 days for 99% of patients. 

Globally, most travel-associated Oropouche cases during the 2024 outbreak were among persons returning from Cuba to the United States, and the data used in this analysis reflect that pattern (*2*,*19*). Before 2024, OROV transmission was not suspected in Cuba, and the population likely had no immunity. By the end of 2024, Cuba had 24,259 suspected cases, of which 626 were confirmed cases (*20*), suggesting high levels of transmission and increased likelihood of repetitive exposure among visitors to Cuba. Compared with travelers to other areas, the estimated incubation period might have been shorter for US travelers to Cuba because they traveled to an area with an active outbreak and likely were exposed, potentially repeatedly, soon after arrival. Going forward, additional data from travelers to other geographic areas can be incorporated to improve the representativeness and precision of the estimate of this Oropouche incubation period.

Prior estimates for the range of the incubation period of Oropouche have included 3–8 days, 4–8 days, and 3–10 days (*3*,*21*,*22*). Our estimate of 1–10 days has a wider overall range than prior estimates, noting symptom onset can occur within 1 day of exposure for 5% of patients. The previous lower estimate of 3 days was based on the symptom onset in a laboratory worker who was exposed orally, and the previous upper estimate of 10 days was documented in a traveler from Brazil who returned to a nonendemic area after visiting the Amazon region (*6*,*10*). The incubation period of 4–8 days was based on an assessment of historical outbreaks in the Brazilian Amazon in the 1960s and 1970s but could not be determined with precision because the exact timing of exposure was unknown for cases in or near endemic areas (*10*). However, evidence suggests that the current viral reassortant replicates faster than the prototype strain, possibly impacting incubation period for infections associated with recent outbreaks (*23*).

In clinical settings, providers should take a thorough history to determine the timing of the patient’s initial symptom onset because relapse of symptoms has been described in up to 70% of Oropouche patients (*24*). Suspicion for Oropouche should be heightened when a patient has a compatible illness within 10 days of returning from an area with active virus transmission. Clinicians should also consider the patient’s underlying immunocompetence, which has been shown to affect incubation period in other arboviral infections, such as West Nile virus (*25*). However, no data are available on the effect of immunosuppression on the Oropouche incubation period. For most patients with clinically compatible illness >2 weeks after return from travel, alternative diagnoses should be considered. If the patient has laboratory evidence of OROV infection, clinicians should evaluate the possibility of local vectorborne transmission or alternative transmission modes.

Although Oropouche is clinically similar to other arboviral diseases, the median incubation period estimated from this analysis (3–4 days; range 1–10 days) is shorter than those for dengue (5–7 days; range 3–10 days), chikungunya (3–7 days; range 1–12 days), and Zika (6–7 days; range 3–14 days) (*17*,*26*,*27*). However, because the estimated ranges of those incubation periods overlap, clinicians should consider potential exposures and other clinical and epidemiologic information when deciding on testing and differential diagnosis.

Knowing the Oropouche incubation period can help direct case investigations and public health response to outbreaks. For example, when OROV infection is confirmed in a traveler >2 weeks after returning to a nonendemic area, public health authorities should consider the possibility of local vectorborne transmission, provided competent vectors are present and seasonality is appropriate, or alternative modes of transmission. Although sexual transmission of OROV has not yet been documented, identification of culturable virus on a day 16 semen sample indicates sexual transmission could occur and should be investigated (*11*). Accurate incubation periods can also help develop criteria for determining the end of an outbreak. A commonly used criterion is twice the longest estimated incubation period without observing any new cases since the last transmission event (*28*). On the basis of the upper limit of the extrinsic incubation period in the vector *Cu. paraensis* midge (8 days) and the upper limit of our estimate of intrinsic incubation (95th quantile of 10 days), a period up to 5 weeks (≈18 days × 2) with no new cases in an area under adequate epidemiologic surveillance could be used to declare the end of an outbreak (*29*).

The first limitation of this study is that the clinically apparent cases included in this analysis potentially biased our results toward shorter incubation periods compared with cases of mild disease. Second, our estimates were based on data from infected travelers, who might have different underlying demographic and medical characteristics compared with nontravelers. Third, our dataset was relatively small, resulting in estimates with less certainty for the upper end of the log-normal distribution (95th and 99th quantiles). Not unexpectedly, 1 extreme observation in our dataset had an exposure window that ended >15 days (99th quantile) before symptom onset. Unusually long lapses between the end of an exposure period and symptom onset could be explained by recall bias because travel duration and symptom onset dates were self-reported by patients. Fourth, data for this project were derived from distinct sources, so event data (e.g., symptom onset) might have been collected differently, impacting the precision of our estimates. Finally, we considered exposures to be vectorborne for cases included in the analysis, but patients with alternative exposures might also have been included in the dataset.

In conclusion, our results indicate that 50% of travelers infected with OROV will develop symptoms within 3–4 days of exposure and 99% will develop symptoms within 15 days. Clinicians and public health responders should evaluate the possibility of alternative modes of transmission (e.g., sexual transmission) or local vectorborne transmission for travelers with Oropouche who have symptoms develop >2 weeks after return from travel. Our estimate of the distribution of the Oropouche incubation period will help clinicians and public health officials develop a differential diagnosis based on the timing of travel-related exposures and inform prevention and control measures.

Appendix Additional information on estimation of incubation period for Oropouche virus disease among 97 probable and confirmed travel-associated cases, 2024–2025.
