# Supplementary material for: Estimation of Incubation Period for Oropouche Virus Disease among Travel-Associated Cases, 2024–2025
Source: Emerg Infect Dis. 2025 Jul;31(7):1337–43. doi: 10.3201/eid3107.250468 (PMC12205460; doi:10.3201/eid3107.250468)
Supplement: Appendix — Additional information on estimation of incubation period for Oropouche virus disease among 97 probable and confirmed travel-associated cases, 2024–2025. [file 25-0468-Techapp-s1.pdf]

Article DOI: https://doi.org/10.3201/eid3107.250468

EID cannot ensure accessibility for supplementary materials supplied by authors. Readers who have difficulty accessing supplementary content should contact the authors for assistance.

Estimation of Incubation Period for Oropouche Virus Disease among Travel-Associated Cases, 2024–2025

Appendix

Appendix Table 1. Comparison of characteristics of probable and confirmed cases identified by CDC (n = 74) vs. GeoSentinel and published literature (n = 23) used in an estimation of incubation period for Oropouche virus disease among travel-associated cases, 2024–2025

Patient characteristic	CDC probable and confirmed cases, n = 74	GeoSentinel and published literature probable and confirmed cases, n = 23	p value
Age group, y			
0–19	3 (4.1)	1 (4.3)	**0.026***
20–39	17 (23.0)	15 (65.2)	
40–59	36 (48.6)	5 (21.7)	
≥60	18 (24.3)	2 (8.7)	
Missing	0	0	
Age, y			W = 9,409;
Mean	48.7	35.7	**p<0.0001†**
Median (IQR)	51 (37–59)	30 (25–41.5)	
Range	11–81	18–71	
Sex			1.0‡
F	41 (55.4)	13 (56.5)	
M	33 (44.6)	10 (43.5)	
Missing	0	0	
Hospitalized			**0.041‡**
No	58 (84.1)	14 (60.9)	
Yes	11 (15.9)	9 (39.1)	
Missing	5	0	
Travel duration, d			**0.00050***
<7	20 (27.0)	1 (4.3)	
7–13	35 (47.3)	3 (13.0)	
14–20	5 (6.8)	12 (52.2)	
21–27	5 (6.8)	3 (13.0)	
≥28	9 (12.2)	4 (17.4)	
Missing	0	0	
Onset during travel			0.48‡
No	34 (45.9)	8 (34.8)	
Yes	40 (54.1)	15 (65.2)	
Missing	0	0	
Exposure period duration (days)			W = 9,409;
Mean	13.7	13.5	**p<0.0001†**
Median (IQR)	7 (5–11)	14.0 (9.0–17.5)	
Range	2–135	2–29	

*Fisher exact test p value.
†Wilcoxon rank sum test p value.
‡χ^2 p value.

Appendix Table 2. Akaike Information Criterion for log-normal, Gamma, and Weibull distributions for different case sets used in an estimation of incubation period for Oropouche virus disease among travel-associated cases, 2024–2025

		Akaike information criterion		
Case set	No.	Log-normal	Gamma	Weibull
Probable and confirmed cases	97	1,300.59	1,305.92	1,308.42
Confirmed cases*	40	543.88	548.07	549.65
2024–2025 probable and confirmed cases	95	1,268.74	1,274.20	1,276.93

*Traveled < 14 d.

Appendix Table 3. Parameters and quantiles of log-normal distribution for different case sets in an estimation of incubation period for Oropouche virus disease among travel-associated cases, 2024–2025

	Confirmed cases* N = 40		2024–2025 probable and confirmed cases N = 95	
Parameter/Quantile	Estimate	95% CI	Estimate	95% CI
Location parameter, μ	3.1	(2.2–4.2)	3.1	(2.5–3.9)
Dispersion parameter, σ	2.0	(1.5–2.4)	1.9	(1.6–2.3)
5th quantile	1.0	(0.4–1.6)	1.1	(0.6–1.5)
50th quantile	3.1	(2.1–4.0)	3.1	(2.4–3.8)
95th quantile	9.3	(5.4–13.2)	9.2	(6.5–11.9)
99th quantile	14.8	(6.9–22.7)	14.4	(8.9–19.9)

* Traveled < 14 d.

Appendix Table 4. Parametric log-normal survival regression models for 97 probable and confirmed travel-associated Oropouche virus disease cases, 2024–2025

Model	Variable (Reference Category)	Coefficient	(95% CI)	SE	P
1	Age	−0.0083	(−0.021 to 0.0047)	0.0066	0.21
2	Sex (Female)				
	Male	0.031	(−0.38 to 0.44)	0.21	0.88
3	Hospitalized (No)				
	Yes	−0.0012	(−0.54 to 0.51)	0.27	0.97
